# What Can Make the Difference Between Chronotypes in Sleep Duration? Testing the Similarity of Their Homeostatic Processes

**DOI:** 10.3389/fnins.2022.832807

**Published:** 2022-03-01

**Authors:** Arcady A. Putilov, Olga G. Donskaya

**Affiliations:** ^1^Laboratory of Sleep/Wake Neurobiology, The Institute of Higher Nervous Activity and Neurophysiology, Russian Academy of Sciences, Moscow, Russia; ^2^Research Group for Math-Modeling of Biomedical Systems, Research Institute for Molecular Biology and Biophysics of the Federal Research Centre for Fundamental and Translational Medicine, Novosibirsk, Russia

**Keywords:** two-process model, simulation, morningness-eveningness, circadian phase, sleepwake cycle, sleep timing, sleep duration, sleep curtailment

## Abstract

The two-process conceptualization of sleep-wake regulation suggests that the biological underpinnings of the differences between morning and evening types in sleep timing and duration might be related to either the circadian process or the homeostatic process or both. The purpose of this report was to test whether morning and evening types might have similar homeostatic processes to achieve such ultimate goal of homeostatic sleep regulation as taking an adequate amount of sleep on free days. Weekend and weekday rise- and bedtimes reported for 50 paired samples of morning and evening types were averaged and simulated with a model of sleep-wake regulation. In morning and evening types of the same age, the homeostatic components of the sleep-wake regulation were found to be identical. Therefore, the difference in the circadian process between chronotypes of similar age can account for the observed differences between them in sleep timing and duration on weekdays and weekends. It was also demonstrated that the model-based simulations might have practical implications for informing an individual about the extent of unrecoverable reduction of his/her sleep on weekdays.

## Introduction

Knowledge of individual differences in the timing of human behavior and physiology seems to be of importance for the optimization of times for taking medications and for attending school, management of fatigue in occupational settings, prediction of performance and risk of accidents on the roads and in unsafe workplaces, diagnosis, and treatment of sleep and circadian rhythm disorders, etc. The recommendations about optimal timing for human activity are often challenged by the lack of information about the causes of the observed profound differences between individuals in the phase characteristics of their circadian rhythms. Additionally, the phase characteristics of such rhythms as the sleep-wake cycle demonstrate notable changes across the lifespan. These changes might cause conflicts between social and biological clocks. For instance, a dramatic weekday sleep loss occurs in late adolescents due to the confrontation between early school start times and their tendency to delay the timing of their sleep on weekends, which, at least partly, is determined by their biology (Crowley et al., 2014).

The publication of Kleitman’s seminal book “Sleep and Wakefulness” (1939) initiated a search for the biological underpinnings of two extremes of the timing of human behavior, known as morningness and eveningness. Given the complexity of the biological mechanisms controlling the phase characteristics of human behavioral and physiological rhythms, valuable insights into the causes of these inter- and intra-individual differences can be provided by mathematical modeling. Model-based simulations can numerically predict the dynamics of the regulatory processes underlying the observed differences between individuals, including the differences between morning and evening (M- and E-) types in these processes. The two-process conceptualization of the mechanisms of sleep-wake regulation (Borbély, 1982) seems to be the major contributor to our current understanding of the causes of differences between individuals in their 24-h sleep-wake pattern. It postulates that the cyclicity of sleep and wakefulness is determined by two regulation processes, a circadian process and a homeostatic process (Borbély, 1982; Daan et al., 1984). The circadian process can be linked to the entrained phase of the circadian pacemaker. It can be measured by tracing such markers of the circadian phase as the times for body temperature minimum and onset of melatonin secretion. The homeostatic process adjusts the intensity and duration of sleep as a function of the duration of prior wakefulness. It can be traced by measuring several spectral electroencephalographic (EEG) indexes during sleep and wakefulness. In particular, Slow Wave Activity (SWA: EEG power density in the 0.75–4.5 Hz range) during non-rapid eye movement sleep was utilized as the spectral EEG marker of the kinetics of sleep homeostasis. It was demonstrated that the exponential function might be applied for the quantitative description of the homeostatic process as the alternations of buildups and decays of SWA in the course of wakefulness and sleep, respectively (Daan et al., 1984; Duffy et al., 1999).

Evidence of the involvement of the circadian process in producing inter- and intra-individual differences in sleep times cannot be questioned. For instance, the results of measurement of body temperature minimum and the onset of melatonin secretion always suggested a 2–3-h difference in the positions of the circadian phase of M- and E-types (Foret, 1982; Kerkhof and van Dongen, 1996; Duffy et al., 1999, 2001; Baehr et al., 2000; Bailey and Heitkemper, 2001; Lack et al., 2009; Paine and Gander, 2016).

Research also provided solid evidence for the contribution of the homeostatic process to the age-associated (intraindividual) differences in sleep timing. For instance, both experiments and simulations confirmed that the kinetics of homeostatic process can be slower in mature adolescents compared to the kinetics in prepubescent adolescents (Jenni et al., 2005; Crowley et al., 2014; Putilov and Verevkin, 2018). Model-based simulations of sleep times showed that the typical changes in sleep timing and duration, from adolescence to old age, can be understood as a consequence of changes in the kinetics of the homeostatic process (Skeldon et al., 2016). Such understanding was supported by experimental research indicating that the age-associated differences cannot be explained by the change in the free-running circadian period (e.g., Czeisler et al., 1999; Kendall et al., 2001; Crowley and Eastman, 2018).

As for the experimental studies of two chronotypes of similar age (intraindividual difference), they suggested that one of the possible sources of a later sleep phase in E-types compared to M-types might be slower kinetics of the homeostatic process (Kerkhof and Lancel, 1991; Lancel and Kerkhof, 1991; Taillard et al., 1999, 2003; Mongrain et al., 2006; Mongrain and Dumont, 2007). However, can such a difference between chronotypes in the kinetics of sleep homeostasis lead to the difference between them in sleep duration? If the result of experimental research shows that the duration of sleep is practically identical in M- and E-types, this implies that the chronotypes are also identical in achieving the ultimate goal of homeostatic sleep regulation, irrespective of chronotype, which is an adequate amount of sleep. Indeed, in accordance with this goal of homeostatic regulation, the vast majority of cited above experiments, M- and E-types were found to be similar to one another in terms of sleep duration, despite significant differences between them in the kinetics of the homeostatic process (e.g., Lancel and Kerkhof, 1991; Taillard et al., 2003; Mongrain and Dumont, 2007).

Even more, if M- and E-types sleep at different circadian phases, there is no way to have identical sleep durations without having different rates of the kinetics of homeostatic processes. The reason for this is because M-types were found to sleep on a later phase of their circadian pacemaker due to a wider phase angle between this pacemaker and sleep (e.g., Duffy et al., 1999, 2001; Baehr et al., 2000; Mongrain et al., 2004; Emens et al., 2009). This was first predicted by simulations (e.g., Putilov, 1995) and then confirmed by the results of experimental research (e.g., Lazar et al., 2015), indicating that the parameters of SWA appear to be modulated by the circadian pacemaker (i.e., by the circadian process). Therefore, to produce similar amounts of sleep in two chronotypes sleeping at different circadian phases, it is necessary to have different rates of homeostatic buildup and decay. For example, when the decay of SWA is quicker on a later circadian phase than on an earlier circadian phase, the duration of decay (sleep) phase on the former phase must be shorter than the duration on the later phase. To make durations identical, the former decay rate must become slower than the later decay rate. Therefore, to clarify whether similar amounts of sleep can be obtained by the chronotypes sleeping at different circadian phases, it is necessary to simulate sleep times in M- and E-types with a model of sleep-wake regulation.

The present study aimed to test the hypothesis of the identity of the homeostatic processes in M- and E-types. We tested whether these processes in two distinct chronotypes are designed for obtaining similar amounts of sleep on free days (i.e., on the days when they are free of any constraints placed on their sleep-wake schedule by the society). The practically important consequence of providing support for the hypothesis of the identity of amounts of sleep in two chronotypes on free days would be a possibility to implement the model-based simulations into the calculation of weekday sleep losses in each of the chronotypes.

## Materials and Methods

The first page of Supplementary Appendix A contains four sleep times (weekday and weekend bed- and rise times) estimated for 50 paired samples of study participants classified into M- and E-types by the authors of published papers. To select these 50 paired samples listed in Supplementary Appendix A, none of the exclusion criteria was applied. In the vast majority of publications, information on four sleep times was found in one of the paper’s tables. If four sleep times were reported for several ages, data on each age were included as separate lines in Supplementary Appendix A. Information on mean age in each sample and the methods of data collection was also included in Supplementary Appendix A. Objective methods (i.e., actigraphy) were used for estimation of sleep times in only seven paired samples, and sleep times were calculated from sleep diaries in four other paired samples. The second page of Supplementary Appendix A contains additional information on the sample sizes and the questionnaires used for the classification of study participants into chronotypes (with the number of items for each of the questionnaires and references). The current sleep times were used to classify into chronotypes only 5 pairs of samples, while, to classify 45 remaining pairs, 9 different versions of diurnal preference scales were applied (see the second page of Supplementary Appendix A).

**FIGURE 1:**
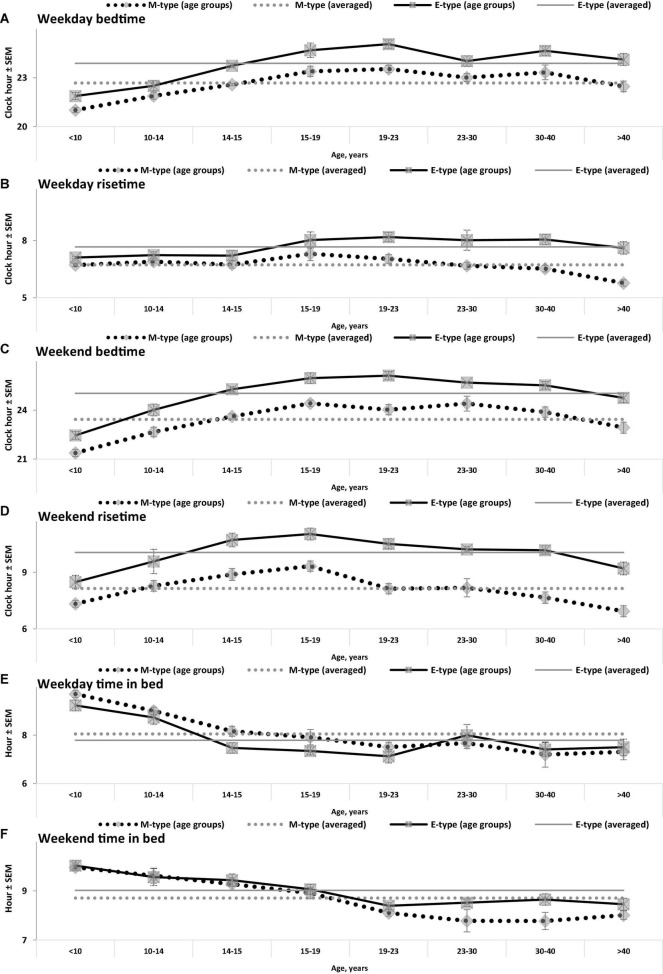
Sleep times on weekdays and weekends in M- and E-types. **(A–F)** Weekday bedtime, weekday risetime, weekend bedtime, weekend risetime, weekday time in bed, weekend time in bed, respectively. Sleep times for the whole set of paired samples (*n* = 50) with mean age of 22.0 years (SEM = 1.8) for mean age of sample and the subsets of samples obtained by grouping into eight intervals with mean ages of sample <10, 10–14, 14–15, 15–19, 19–23, 23–30, 30–40, and >40 years (*n* = 6, 5, 7, 7, 7, 7, 5, and 6, respectively). See also the list of samples in Supplementary Appendix A and SEM for sleep times averaged over 50 samples in Table 1.

**FIGURE 2:**
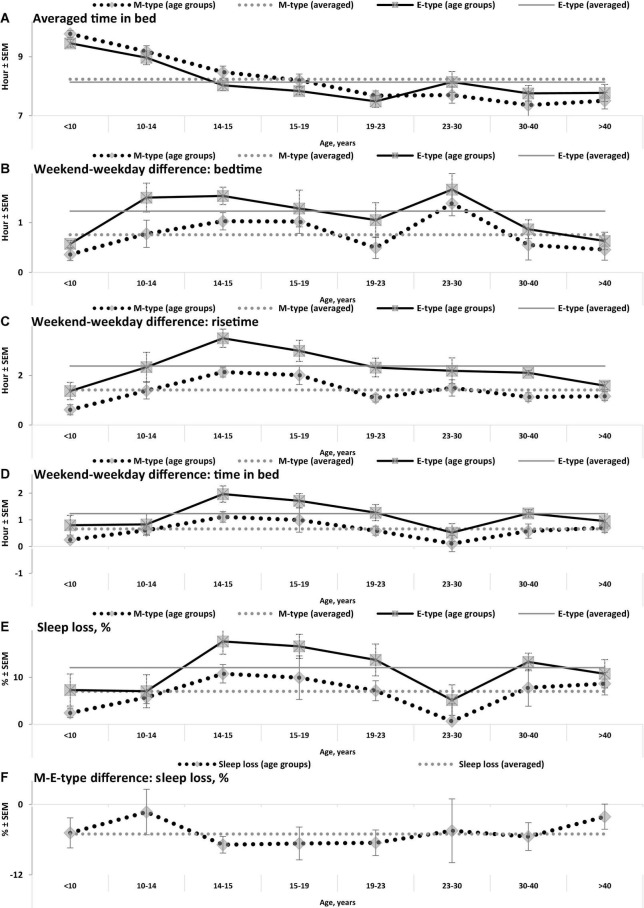
Averaged time in bed, weekend-weekday differences, and sleep loss in M- and E-types. **(A–F)** Weekday bedtime, weekday risetime, weekend bedtime, weekend risetime, weekday time in bed, weekend time in bed, respectively. See SEM for averaged over 50 samples sleep times in Tables 1, 3, and see also other notes in the notes to Table 1 and in the legend to Figure 1.

**FIGURE 3:**
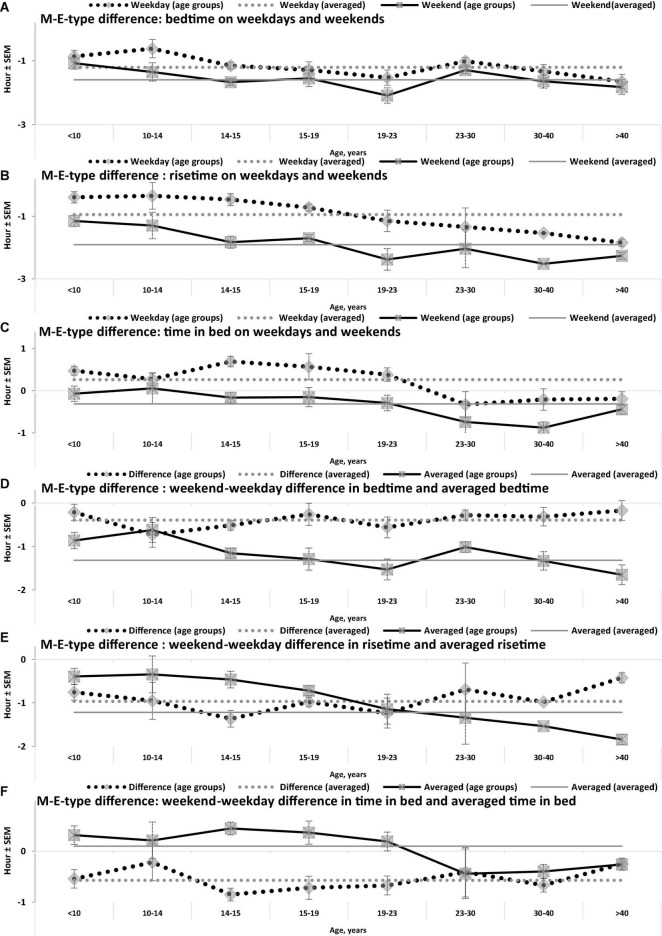
Difference between M- and E-types in bed- and rise-times and in time in bed. **(A–F)** Weekday bedtime, weekday risetime, weekend bedtime, weekend risetime, weekday time in bed, weekend time in bed, respectively. See SEM for averaged over 50 samples sleep times in Tables 1, 3, and see also the notes to Table 3 and the legend to Figure 1.

The paired samples were assigned to eight age groups to test the significance of age-associated changes in the differences between M- and E-types in sleep times with one-way ANOVAs (Figures 1–3), and paired Student’s *t*-test was employed for the comparison of mean sleep times obtained for M- and E-types by averaging over 50 samples (Table 1). The statistical tests were performed using the Statistical Package for the Social Sciences (SPSS 23, IBM, Armonk, NY, United States).

**TABLE 1:** Sleep times averaged over 50 samples of M- and E-types.

Sleep	Time	M-type		E-type		“Age”: *F*_7/42_	
		Mean	SEM	Mean	SEM	M-type	E-type
Bed-time, clock h or h	Weekday	22.677	0.14	23.882	0.174	11.674***	14.276***
	Weekend	23.435	0.164	25.031	0.182	12.394***	23.186***
	Difference	0.757	0.086	1.149	0.103	2.471*	2.422*
	Averaged	22.893	0.142	24.210	0.170	13.591***	19.541***
Rise-time, clock h or h	Weekday	6.726	0.091	7.669	0.117	4.452**	2.208
	Weekend	8.142	0.147	10.049	0.163	6.453***	5.730***
	Difference	1.416	0.108	2.380	0.162	4.655**	3.588**
	Averaged	7.131	0.098	8.349	0.109	5.592***	3.371**
Time in bed, h	Weekday	8.049	0.143	7.787	0.135	9.777***	7.020***
	Weekend	8.708	0.140	9.019	0.110	11.078***	7.111***
	Difference	0.659	0.092	1.232	0.124	1.927	2.547*
	Averaged	8.237	0.136	8.139	0.115	11.879***	8.858***
Sleep	loss, %	7.016	1.015	12.065	1.190	1.603	2.236

**FIGURE 4:**
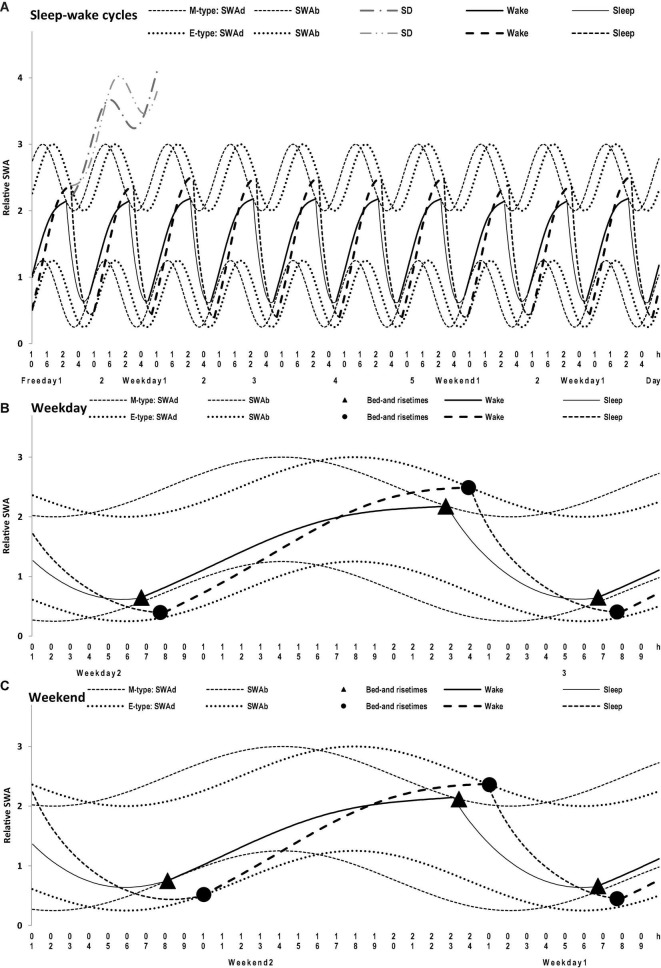
Simulations of a sequence of 10 sleep-wake cycles in M- and E-types. **(A)** The whole sequence of 10 sleep-wake cycles, 2 last free days (e.g., at the end of hypothetical vacation), the following week consisting of 5 weekdays and 2 weekends, and the 1st weekday of the next week; **(B,C)** The weekday and weekend cycles, respectively, on 1.5-day intervals of the whole sequence of 10 sleep-wake cycles **(A)** with mean bed- and rise-times from Table 1. SWAd and SWAb: the highest expected buildup and the lowest expected decay of relative SWA, respectively; DS: Deprivation from Sleep leads to a further buildup of SWA above the highest buildup (i.e., the effect of prolonging wakefulness beyond bedtime that is usually interpreted as “accumulation of sleep dept” with the expected “paying back” during the following recovery sleep episode); Wake and Sleep: two phases of the sleep-wake regulating process that is, in the simulations, exponential buildups and decays of SWA, with the assumption that the parameters of these exponential buildups and decays are modulated by sine-form function over a 24-h period (i.e., the circadian term). See the parameters of the model (1) applied for these simulations in Table 2.

**FIGURE 5:**
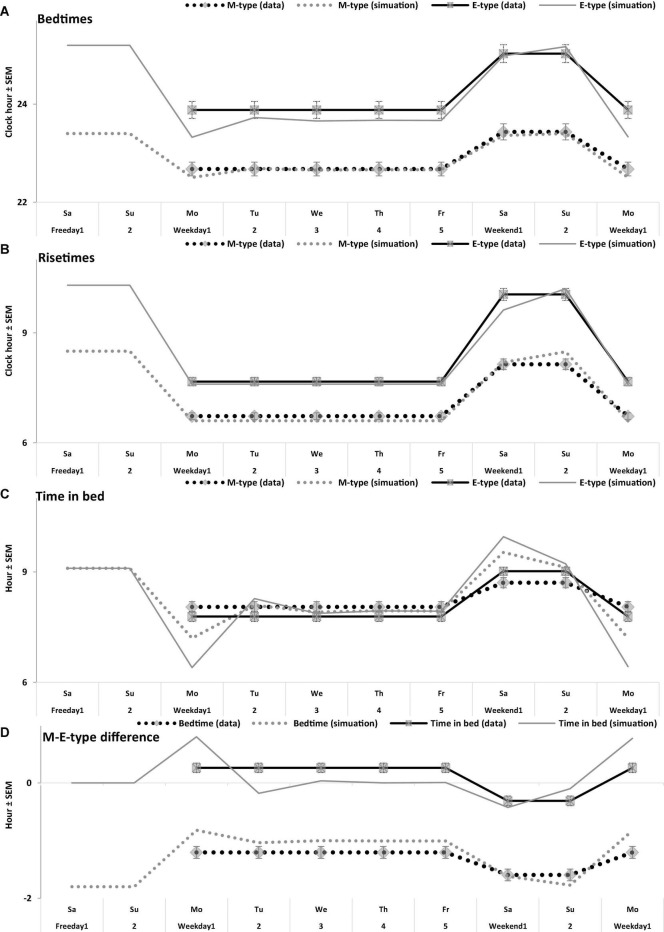
Time course of sleep times of M- and E-types on the interval of 10 sleep-wake cycles. **(A–D)** Bedtime, risetime, time in bed, difference between M- and E-types in bedtime and time in bed, respectively. Comparison of the averaged data on sleep times for 5 weekdays and the following 2 weekend days with the simulated sleep times for the whole sequence of 10 sleep-wake cycles (Figure 4A). The parameters of the model are listed in Table 2 and compared with the averaged data in Table 3. Mean sleep times for 50 samples are shown in Figures 1–4 and included in Table 1.

Mean and SEM: sleep time obtained by averaging over 50 samples (see Supplementary Appendix A) and Standard Error of this Mean; Difference: difference in sleep time between weekend and weekday; M-E-type difference: difference between two chronotypes; Averaged: weekly averaged sleep time; Sleep Loss: percentage of sleep lost due to the advance of wakeups on weekdays, calculated as: 100 × Weekend-Weekday Difference in Risetime/(24 + Weekend Risetime – Weekday Bedtime); “Age”: F_7/42_: F-ratio for the main effect of independent factor “Age” (one-way ANOVA); *p < 0.05, **p < 0.01, and ***p < 0.001 for F_7/42_; Mean age calculated for 50 paired samples was 20.0 years with SEM = 1.8 years; See also comparisons with simulations in Figure 5 and sample-averaged sleep times in Figures 1, 4, 5.

**TABLE 2:** Parameters of the model applied for simulations of sleep times in M- and E-types.

	Simulation parameters	*Previous*	50 paired samples		
			M-type	E-type	Difference
Initial	*t2* (bedtime), clock h	23.00	23.40	25.20	–1.80
times for	*t1* (risetime), clock h	7.00	8.50	10.30	–1.80
two phases	Weekday risetime, clock h	–	6.60	7.60	–1.00
Parameters	*T*_*b*_ (time constant of buildup phase), h	27.04	26.63	22.67	3.95
of phases of	*T*_*d*_ (time constant of decay phase), h	1.95	2.75	2.07	0.68
buildup and	SWA_*l*_ (lower asymptote), relative SWA	0.70	0.70	0.70	0.00
decay	SWA_*b*_ (lowest decay), relative SWA	0.75	0.76	0.76	0.00
	SWA_*d*_ (highest buildup), relative SWA	2.50	3.00	3.00	0.00
	SWA_*u*_ (upper asymptote), relative SWA	4.50	5.50	5.50	0.00
Parameters	*A* (circadian amplitude), relative SWA	0.50	0.50	0.50	0.00
of sine	φ_*max*_ (circadian peak), clock h	15.00	14.10	17.90	–3.80
wave-form	Phase angle between φ_*max*_ and *t2*, h	8.00	9.30	7.30	2.00
circadian	τ (entrained circadian period), h	24.00	24.00	24.00	0.00
modulation	*k* (twofold increase of the circadian term)	2.00	2.00	2.00	0.00

*Parameters of the model (1) applied for simulations of sleep times in M- and E-types illustrated in Figures 4, 5. Previous: the parameters of this model were previously derived in Putilov (1995) by using data on sleep duration after extended wakefulness and on SWA in naps and extended sleep episodes (mean SWA = 1 in baseline night episode). 50 paired samples: this study simulation; Difference: difference between two chronotypes. A slight modification of the parameters was necessary to account for the differences between initial and present study data (e.g., relatively higher SWA levels and longer sleep duration for younger ages in the present study samples). Moreover, bed- and rise-times and circadian phases were proposed to be set earlier and later to account for the differences between M- and E-types, respectively (1.8- and 3.8-h M-E-type difference, respectively). It was also suggested that the difference in Weekday risetime is equal to 1 h (6.6 and 7.6 h for M- and E-types, respectively).*

**TABLE 3:** Difference between M- and E-types in sleep times, averaged over 50 samples.

Sleep	Time	M-E-type difference: data			Simulation	Discrepancy	“Age” *F*_7/42_
		Mean	SEM	*t*_49_			
Bed-time, h	Weekday	–1.205	0.104	−11.580***	–0.975	–0.230	1.311
	Weekend	–1.596	0.102	−15.615***	–1.697	0.101	1.347
	Difference	–0.392	0.071	−5.521***	–0.722	0.330	0.900
	Averaged	–1.317	0.098	−13.376***	–1.181	–0.136	1.361
Rise-time, h	Weekday	–0.943	0.117	−8.059***	–1.000	0.057	4.141**
	Weekend	–1.907	0.124	−15.383***	–1.576	–0.331	2.206
	Difference	–0.965	0.101	−9.591***	–0.576	–0.389	1.302
	Averaged	–1.218	0.110	−11.055***	–1.165	–0.053	3.950**
Time in bed, h	Weekday	0.262	0.083	3.165**	0.133	0.129	3.963**
	Weekend	–0.311	0.085	−3.665**	–0.263	–0.048	1.750
	Difference	–0.573	0.095	−6.038***	–0.396	–0.177	0.713
	Averaged	0.098	0.071	1.377	0.020	0.078	4.573**
Sleep	loss, %	–5.049	0.940	−5.368***	–4.547	–0.502	0.570

Difference: difference in sleep time between weekend and weekday; Averaged: weekly averaged sleep times; Sleep Loss: percentage of amount of sleep lost due to the advance of wakeups on weekdays; M-E-type difference: data: difference between two chronotypes in data reported in Table 1; Simulation: simulation of data with parameters listed in Table 2; Discrepancy: difference between simulation and data; “Age,” F_7/42_: F-ratio for the main effect of independent factor “Age” for a difference between chronotypes in Sleep time (one-way ANOVA); Mean and SEM: mean for Difference between two chronotypes in Sleep time obtained by averaging over 50 samples and Standard Error of Difference; t_49_: paired student’s t-test for comparison of paired samples of M- and E-types; **p < 0.01, ***p < 0.001 for t_49_ or F_7/42_. See also comparisons of data and simulations in Figure 5 and the sample-averaged differences in sleep times in Figures 2–5.

The average weekday and weekend sleep times obtained for M- and E-types (Table 1) were simulated with a model of sleep-wake regulating processes (Putilov, 1995) that postulates the modulating influence of the circadian process on the parameters of SWA, a marker of the homeostatic process. In this model, *t1* and *t2* are the initial times for the buildup and decay phases of the 24-h sleep-wake cycle (i.e., the rise- and bedtimes on free days, respectively). The process of sleep-wake regulation, *X*(*t*), is simulated using the following equations:


(1a) $$X\left(t\right)=\left[X_{u}+C\left(t\right)\right]-\left\{\left[X_{u}+C\left(t\right)\right]-X_{b}\right\}*e^{-\left(t-t1\right)/\left[Tb-k*C\left(t\right)\right]}$$

(1b) $$X\left(t\right)=\left[X_{l}+C\left(t\right)\right]-\left\{X_{d}-\left[X_{l}+C\left(t\right)\right]\right\}*e^{-\left(t-t2\right)/\left[Td-k*C\left(t\right)\right]}$$

where


(2) $$C\left(t\right)=A*sin\left(2\pi *t/\tau +\varphi_{0}\right)$$

is a periodic function with a period τ assigned to 24 h. This term (2) represents the modulating effect of the circadian process on the parameters of the homeostatic process represented by the time course of relative SWA (Figure 4). All parameters of the model applied for the simulations of sleep times in M- and E-types are listed in Table 2. A difference between chronotypes in the phase angle between the circadian pacemaker and sleep was suggested to reach 3.8 h with M-types sleeping at a later phase of their circadian pacemaker. The difference in their sleep times on free days was proposed to be close to the empirically obtained mean value for weekends, 1.8 h (Tables 1–3). We also proposed a 1-h difference in weekday risetime between M- and E-types that is also close to the empirically obtained mean value (Tables 1–3). Table 3 provides the estimates of the empirically obtained differences between chronotypes and the estimates of discrepancies between these and simulated values.

## Results

While the sleep times obtained for any of two chronotypes drastically changed throughout the lifespan (Figure 1), some of the differences between M- and E-types in these times did not change (Figure 3 and Table 3). The statistically significant changes were limited to the weekend risetime and weekend time in bed, as well as to the weekly averaged risetime and weekly averaged time in bed for which the major contributors are the weekday risetime and weekday time in bed (Figures 2, 3 and Table 3). Undoubtedly, the result suggested that the biologically determined differences between chronotypes do not change with advancing age, and significant age-associated changes in the difference in weekday sleep cannot be explained by the biological differences between chronotypes. Instead, they can be explained by the age-associated weakening of the social constraints placed on sleep-wake schedule of E-type participants (i.e., these constraints seem to be strict for school students with E-type but less strict for working adults with E-types).

Irrespective of age, weekend-weekday difference in bedtime, risetime, and time in bed were smaller in M-types than in E-types (Figure 2 and Table 3). Sleep loss was also smaller in M-types than in E-types (Figure 2 and Table 3). Finally, weekday time in bed was longer in M-types, whereas weekend time in bed was longer in E-types (Figure 3 and Table 3). As suggested by the results reported in Table 3 and illustrated in Figures 4, 5, such differences between M- and E-types were predicted by the simulations based on the assumption that sleep durations in these types on free days are equal (i.e., 9.00 h for both in Table 2). Notably, the simulations predicted only a tiny difference in favor of M-types in weekly averaged time in bed (0.020 h), and the analysis of empirical data also provided a similarly small and same-directional difference in weekly averaged time in bed in favor of M-types (0.098 h with standard error of 0.071 h).

The simulations explain why all these differences between chronotypes emerge despite the identity of their time in bed on free days (Table 2). Compared to M-types, E-types slept less on weekdays and more on weekends because sleep reduction on weekdays was larger in E-types to be extended more on weekends (Figures 5C,D). In other words, this was a model-predicted consequence of a smaller difference between E-and M-types in the advancing weekday shift of their bedtime compared to a larger difference between them in the advancing shift of risetime (Figures 5A,B). As a result of the differences between chronotypes caused by the transition to weekdays, the difference between them during the following delaying shift of risetime on weekends has to be, in turn, relatively larger than the difference in in the shift of bedtimes (Figures 5A,B).

## Discussion

Since the pioneering work of Kleitman (1939), a search for the biological mechanisms underlying the differences between M- and E-types has remained an intriguing topic for many studies in the field of sleep science and chronobiology. In accord with the two-process conceptualization of sleep-wake regulation processes (Borbély, 1982; Daan et al., 1984), the biological underpinning of the observed differences between M- and E-types in terms of sleep time might be explained by the difference in either the circadian process or the homeostatic process or both (e.g., Kerkhof and Lancel, 1991; Lancel and Kerkhof, 1991; Taillard et al., 1999, 2003; Mongrain et al., 2006; Mongrain and Dumont, 2007). Previous studies simulating sleep times using two-process models of sleep regulation have concluded that there might not be a need to consider differences in the circadian process in explaining and predicting intraindividual (not interindividual) differences in sleep timing across the lifespan (Skeldon et al., 2016; Putilov and Verevkin, 2018). The sleep times reported in the literature for 50 paired samples of M- and E-types of the same age were analyzed and simulated with a model, suggesting the possibility of circadian modulation of the homeostatic process. Our simulations support the hypothesis of the sleep homeostatic processes in two distinct chronotypes of the same age.

The ultimate goal of homeostatic regulation, as a way of enabling people to have an adequate amount of sleep on free days, was achieved by the study participants irrespective of their chronotype. Overall, the simulations of sleep times in M- and E-type people of the same age indicate that the homeostatic processes of sleep regulation might be identical. This result provides further support for the results of several other experimental studies (e.g., Lancel and Kerkhof, 1991; Taillard et al., 2003; Mongrain and Dumont, 2007), which indicated that M- and E-types were similar one to another in terms of sleep duration despite significant differences in the kinetics of their homeostatic process.

To our knowledge, this is the first analysis of sleep times in a hundred samples of M- and E-types from various age groups, some of the obtained results are of special interest for practical reasons.

(1) Using clock times reported for sleep episodes as criteria for the classification of people into M- and E-types cannot be recommended. It seems that sleep of M-types from one age group occurs during clock times that are not earlier, but later than clock times for sleep of E-types from some other age groups (e.g., when the group of late adolescence age are compared to the groups of middle adulthood, childhood, and early adolescence age).
(2) Despite this drastic age-associated change in sleep timing and despite the significant age-associated differences in social constraints imposed on weekday sleep-wake schedules, the biological difference between M- and E-types in sleep timing remains stable across ages. It seems that this difference is determined exclusively by the difference between them in the circadian process.
(3) E-types do not always sleep less on weekdays than M-types. In the analyzed dataset, the opposite is true for E-types of older ages who, when compared to E-types of younger ages, are experiencing less strict social constraints placed on their sleep-wake schedule. For instance, when E-types from study participants were working adults, they appeared to manage to profoundly delay their weekday wakeups compared to M-types from the same study, but when these were university and, especially, school students, they had to arrive at their university/school not later than M-types.
(4) Estimates as a percentage of weekday sleep loss rather than weekly averaged sleep duration accurately reflect the reduction of weekday sleep duration and indicate a larger weekday sleep loss in E-types than M-types. For instance, the latter estimate suggested similarities of mean sleep duration in two chronotypes while the former estimate indicated that, irrespective of age, E-types experienced somewhat larger weekday sleep losses than M-types due to a larger weekend-weekday differences in sleep timing.
(5) The results did not suggest that E-types profoundly differ from M-types in the amount of sleep lost on weekdays. Moreover, since the difference between two chronotypes in estimates such as percentage of weekday sleep loss remains stable across ages, weekday sleep losses in late adolescents with E- and M-types differ from one another to a larger extent than sleep losses in middle age adults with E- and M-types only in absolute terms.
(6) Both E-types and M-types are vulnerable to weekday sleep losses, especially at older ages when E-types are not experiencing very strict social constraints placed on their sleep-wake schedule. Since M-types can tolerate early morning wakeups better than E-types, they do not care as much as E-types about the consequences of early weekday wakeups for their health. Therefore, E-types of older ages might not be considered more vulnerable than M-types to the aversive health effects of losing sleep on weekdays.
(7) None of the minutes of sleep lost on weekdays can be caught up on weekends. The simulations of weekday sleep times in M- and E-types suggested that their SWA on these days did not build up above the upper threshold set by the homeostatic sleep-wake regulator. In other words, there is nothing to be “paying back” on weekends when, in the previous weekdays, there is no “accumulation of sleep debt” represented in the model by a buildup of SWA above this threshold.
(8) Therefore, although E-types can sleep longer than M-types on weekends, this cannot be explained by their capability to compensate for sleep lost on weekdays by getting extra sleep on weekends. Their weekend sleep was similar to the weekend sleep in M-types, and it was very close in duration to the duration of adequate sleep of any of the chronotypes on free days. In other words, E-types are not tacking on an extra hour or two of sleep a night on weekends, but they are getting sleep of near normal duration (i.e., this duration is close to the duration set by the homeostatic sleep-wake regulator on free days).
(9) Although the simulations suggested a large difference between M- and E-types in the circadian phase and despite free access to artificial lighting on the weekend evenings, the circadian pacemaker in all chronotypes remains entrained to external time cues throughout a week, and, in turn, the sleep-wake cycle remains in sync with this circadian pacemaker. Although the back and forth shifts of bed- and risetimes on weekdays and weekends look like a weekend-weekday difference in sleep timing, this difference has nothing to do with the shifts of phases or with the changes in phase angle. In other words, neither the circadian phase nor sleep phase nor the phase angle between them shifts throughout the week, and, therefore, the circadian system cannot be disrupted by early wakeups on weekdays. The aversive effects of early weekday wakeups exist, but they are limited to the unrecoverable reduction of weekday sleep.

Such results might have practical implications. For instance, for any individual of certain age and chronotype, the quantitative predictions of adequate amount of sleep required for avoiding adverse health consequences of weekday sleep loss can be made.

There are several limitations affecting these simulations of the sleep-wake regulation processes, which relied exclusively on bed- and rise-times as model inputs. One of the major limitations is the absence of any additional data on the markers of sleep intensity and circadian phases, such as changes in SWA levels during sleep and clock times for body temperature minimum or onset of melatonin secretion. Moreover, the objective methods (i.e., actigraphy) were applied for measuring sleep times only in a minor fraction of the analyzed samples (see Supplementary Appendix A). An additional disadvantage of our simulations was in using bed- and rise-times as the input of the model instead of times for sleep onset and offset. However, since sleep onset and offset were mostly provided through self-reporting, this calculation included some other subjective reports, such as the self-assessment of sleep latency, which are of even more questionable accuracy than the self-reporting of bed- and rise-times. It should also be mentioned that there were several other sources of variation in the estimates of sleep times that cannot be excluded or accounted for in the present analysis, i.e., different questionnaire tools were used for the classification of chronotypes by the authors of the published studies. Their studies were conducted in different countries with different sleep-related customs, during different seasons, and under different natural and artificial light-dark conditions. Furthermore, we cannot account in our simulations for the interplay between the biological sleep regulators and various psychological factors contributing to differences between chronotypes in sleep timing. Only average data were simulated because the number of samples in each of the 8 age groups was not big enough to test whether the model predictions remain practically the same for any of these groups.

Moreover, we simulated only sleep-wake cycles and did not consider other chronobiological differences between the two chronotypes. In particular, the simulations did not account for the differences between M- and E-types in the alertness-sleepiness rhythm. Despite a common-sense view that daily fluctuations of alertness-sleepiness level are simply a reflection of the human sleep-wake cycle, these fluctuations can be regarded as an example of a “strong” (rigid) circadian rhythm, such as the diurnal patterns of core body temperature and melatonin secretion. A “strong” circadian rhythm is characterized by a narrow range of entrainment that is the range of synchronizer’s periods to which a given rhythm can entrain. Unlike a weaker (lax) rhythm (i.e., the sleep-wake cycle), such a “strong” rhythm cannot be entrained by a time giver with a much longer or much shorter period (Pittendrigh and Daan, 1976; Granada et al., 2013). The experimental results indicate that the range of entrainment of alertness-sleepiness rhythm is even narrower than that of the core body temperature rhythm (Folkard et al., 1985). Therefore, the simulations of the alertness-sleepiness rhythm could require somewhat different and more advanced models than simulations of the sleep-wake cycle. At least one additional process is usually included in a model of fluctuations of alertness-sleepiness level (e.g., Åkerstedt and Folkard, 1997). For instance, since alertness demonstrates a gradual declining trend from one day to another in the course of prolonged wakefulness, this trend might be accounted for by postulating an additional change in, at least, one of the asymptotes that cannot be, as it is suggested in Eq. 1, a constant throughout the buildup (wake) phase of the cycle (Putilov et al., 2014, 2015, 2019). While the number of samples with sleep times is already sufficient for performing the present statistical analysis and simulations, the published data on “strong” rhythms in M- and E-types remains scarce. The accumulation of such data in future studies would allow the inclusion of simulations (1,2) and empirically derived (not hypothetical) estimates of the differences between chronotypes in the circadian phase and phase angle between this phase and sleep times on free days.

## Conclusion

We tested the hypothesis of the general similarity of the homeostatic processes in morning and evening chronotypes. The homeostatic processes in the two chronotypes were found to be similar, at least, in terms of achieving the ultimate goal of homeostatic regulation, such as getting an adequate amount of sleep on free days. Therefore, in future studies of the mechanisms underlying the difference between chronotypes, it would be sufficient to evaluate the difference between them in the circadian process. In practical terms, the model-based simulations can be applied for the quantitative prediction of sleep losses associated with early weekday wakeups.

## Data Availability Statement

The original contributions presented in the study are included in the article/Supplementary Material, further inquiries can be directed to the corresponding author.

## Ethics Statement

The studies involving human participants were reviewed and approved by the Ethic Committee of the FRC FTM. The patients/participants provided their written informed consent to participate in this study.

## Author Contributions

AP: conceptualization, data curation, formal analysis, funding acquisition, investigation, methodology, project administration, resources, software, supervision, validation, visualization, writing – original draft, and writing – review and editing. OD: funding acquisition, project administration, resources, writing – review and editing. Both authors contributed to the article and approved the submitted version.

## Conflict of Interest

The authors declare that the research was conducted in the absence of any commercial or financial relationships that could be construed as a potential conflict of interest.

## Publisher’s Note

All claims expressed in this article are solely those of the authors and do not necessarily represent those of their affiliated organizations, or those of the publisher, the editors and the reviewers. Any product that may be evaluated in this article, or claim that may be made by its manufacturer, is not guaranteed or endorsed by the publisher.
